# Nanocarbon as a Quantum Material for Biointerfaces and Magnetic Platforms in Theranostic Biomedicine in Oncology: A Critical Review

**DOI:** 10.3390/bios16090482

**Published:** 2026-09-01

**Authors:** Priscila M. Galdino, Barbara R. Geraldino, Nilséia A. Barbosa, Fernando M. Araújo-Moreira

**Affiliations:** Nuclear Engineering Department, Instituto Militar de Engenharia (IME), Praça General Tibúrcio, 80, Urca, Rio de Janeiro 22290-270, Brazil; priscila.magalhaes@ime.eb.br (P.M.G.); barbara.geraldino@ime.eb.br (B.R.G.); nilseia.barbosa@ime.eb.br (N.A.B.)

**Keywords:** nanocarbon, graphene, carbon nanotubes, carbon dots, oncology biosensing, magnetic-assisted biosensing, biointerfaces, analytical validation, magnetic carbon, drug delivery, translational nanomedicine

## Abstract

Nanostructured carbon materials are low-dimensional systems relevant to oncology biosensing, with their utility arising from an electronic structure coupled to defect and edge states, a charge-transfer behavior and optical response that report molecular binding, an interfacial chemistry governing contact with the analyte, and, in selected cases, magnetic properties enabling manipulation and readout. In functional terms, these behaviors trace to specific quantum-relevant features—quantum confinement, edge and defect states, and the resulting size-dependent optical and charge-transfer responses—rather than to a generic quantum-material designation. This critical review treats nanocarbons as engineered biointerfaces whose performance is set by how the carbon surface behaves in biological fluid, how recognition chemistry is anchored, and how the binding event is transduced, with magnetic responsiveness, stability, reproducibility, and fabrication control as decisive constraints. The analysis separates three material classes—non-magnetic nanocarbon sensors, hybrid carbon–magnetic systems, and defect-associated or potentially metal-free magnetic carbons—while grading evidence as direct, adjacent, comparator-derived, or prospective. Directly, graphene, carbon nanotubes, carbon dots, graphene quantum dots, and magnetic carbon hybrids serve in electrochemical, optical and fluorescent, field-effect, and magnetic-assisted formats. Clinical translation, however, remains constrained by biofouling, protein-corona formation, matrix interference, unstable functionalization, batch variability, incomplete standardization, and scarce validation in real samples and patient cohorts. Metal-free or defect-associated magnetic carbons therefore warrant caution, remaining prospective platforms until the preservation of magnetic response, reproducible functionalization, matrix compatibility, safety, and measurable analytical advantage are directly demonstrated.

## 1. Introduction

Nanostructured carbon materials have become important components of biomedical engineering because their dimensionality, surface chemistry, electronic structure, optical response, and interfacial reactivity can be tuned across distinct material families. These families span graphene-based materials such as graphene oxide and reduced graphene oxide, as well as carbon nanotubes, carbon dots, graphene quantum dots, nanodiamonds, fullerenes, and defect-engineered carbons [1,2,3,4,5,6,7,8,9]. In oncology, these materials are especially relevant at the biointerface, where nanoscale carbon surfaces interact with proteins, biological fluids, recognition ligands, immune components, tumor cells, extracellular matrices, and complex microenvironmental gradients. These interactions underpin biosensing- and imaging-compatible detection in oncology; drug delivery and theranostic designs are considered only in an adjacent context (Section 7) and are not the focus of this review.

Material properties alone do not determine biomedical relevance. The very features that make nanocarbons attractive—large surface area, conductivity, optical responsiveness, adsorption capacity, and chemical functionalizability—can generate sensitive signals and support molecular recognition, yet each can also work against the device: surfaces foul and acquire a protein corona, particles aggregate, ligand accessibility shifts, biodistribution becomes uncertain, the immune system intervenes, toxicity appears, and performance drifts from batch to batch [10,11,12,13,14,15,16,17]. This double-edged matter is most important for oncology biosensors, whose performance must hold up not in buffer but in serum, plasma, urine, saliva, cell lysates, tissue extracts, and tumor-associated fluids. It is against those matrices that the decisive metrics are read: limit of detection and selectivity, linear range and signal stability, resistance to matrix interference, regeneration and reproducibility, and comparison with established platforms [5,6].

Calling nanocarbons quantum-relevant demands precision. The term is used here in a functional sense: it points to features of low-dimensional or defect-engineered carbon—quantum confinement, edge and defect states, the electronic density of states, charge-transfer behavior, size-dependent optical responses, spin-related effects, and defect-associated magnetism—that can shape how a device transduces signal, fluoresces, behaves electrochemically, responds to a magnetic field, or interacts at the nano-biointerface. The framing bears most directly on magnetic and hybrid nanocarbon platforms, where heterogeneity in composition, mechanism, and maturity feeds through into characterization requirements, safety interpretation, reproducibility, and regulatory expectations [6,8,9].

Within this framework, the review prioritizes carbon-based and magnetically responsive biointerfaces for oncology biosensing. Magnetic nanocarbon platforms are considered when magnetic responsiveness may improve analyte capture, preconcentration, matrix washing, localization, or integration with electrochemical, optical, fluorescent, field-effect, or magnetic-assisted readouts. Hybrid carbon–metal-oxide constructs, magnetic graphene derivatives, magnetic carbon dots, carbon–SPION architectures, and defect-associated or potentially metal-free magnetic carbons are treated according to evidence level and intended biosensing function.

### 1.1. Material Class, Scope, and Terminology

The magnetic nanocarbon platforms considered in this review range from well-characterized hybrids to largely conceptual architectures. Within that range, metal-free or defect-associated magnetic carbon raises a specific question: whether magnetism carried by the carbon lattice itself can support a biosensing interface. MAGUS enters the review as a worked example of that architecture, not as its subject. It is used as a prospective case study and is not evaluated as a commercial product or as a clinically validated diagnostic, therapeutic, theranostic, or biosensing technology. The name refers to the “MAgnetic Graphite Universal System”, a magnetic carbon/graphite architecture proposed in prior work by some of the present authors [18,19]; that work suggests it could, in principle, be functionalized with ligands, radionuclides, antibodies, or other targeting components, though such functionalizations have not yet been experimentally demonstrated. The available literature therefore supports discussing MAGUS as an architectural hypothesis and a comparator case, rather than as a mature biomedical platform.

Three related but non-equivalent terms are used throughout, from the general to the specific. “Defect-associated magnetic carbons” denotes the broad material class in which magnetic behavior arises from structural defects, edge states, vacancies, disorder, or related electronic features [18]. “MAGUS-like platforms” denotes the narrower set of prospective systems that combine such magnetic or defect-associated carbon cores with functional biological, chemical, or therapeutic components. “MAGUS” denotes only the single named instance described in the present authors’ prior work [18,19]. The object of this review is the class; MAGUS appears as one case within it.

### 1.2. Evidence Boundaries and Translational-Readiness Framework

Because the nanocarbon oncology-biosensing literature spans materials at very different levels of maturity, this review evaluates each claim against an explicit evidence hierarchy to prevent analytical feasibility from being mistaken for clinical utility. The hierarchy has four tiers. Direct evidence comes from a specific platform itself, or from a compositionally and functionally equivalent material. Adjacent nanocarbon evidence includes graphene, graphene oxide, reduced graphene oxide, carbon nanotubes, carbon dots, graphene quantum dots, and related carbons. Comparator magnetic evidence comes from SPIONs, magnetic graphene hybrids, or other magnetic systems. Prospective inference rests on design rationale or extrapolation. On their own, adjacent and comparator evidence establish mechanistic plausibility and functional benchmarks but do not validate a specific platform.

Readiness follows the same logic. A laboratory demonstration establishes analytical feasibility under controlled conditions; translational relevance demands more—performance in real biological matrices, reproducible fabrication, stability, scalability, and a direct comparison with established methods—since high sensitivity alone does not secure adoption [20]. Proof of concept is thus necessary but not sufficient for any claim of diagnostic, biosensing, or clinical maturity. Before such a claim holds, a candidate must still pass physicochemical standardization, matrix testing, independent replication, and, where applicable, regulatory validation [21].

Because direct biosensing and oncology evidence on MAGUS is not yet available, adjacent nanocarbon and comparator magnetic literature is used only to define benchmarks and validation requirements for MAGUS-like platforms, not to raise their readiness level or substitute for direct validation.

## 2. Review Approach and Evidence Synthesis

This article is a critical narrative review supported by structured evidence mapping. It is not a systematic or scoping review and does not claim exhaustive retrieval, pooled quantitative synthesis, or formal risk-of-bias assessment. This approach was selected because nanocarbon platforms differ substantially in composition, dimensionality, surface chemistry, functionalization strategy, magnetic behavior, biological interface, and intended application, limiting the interpretability of pooled synthesis at the current stage of the field. The review instead aims to identify translational barriers, distinguish demonstrated evidence from comparator-based plausibility, and define the evidence requirements that an early-stage magnetic nanocarbon platform would need to meet in order to progress from proof of concept toward analytical, biological, and clinical maturity.

The evidence base included peer-reviewed original articles, reviews, methodological studies, regulatory documents, recognized standards, and clinical registry records. Searches were conducted in PubMed/MEDLINE, Scopus, Web of Science, and ClinicalTrials.gov, as well as FDA guidance documents and ISO standards. Search strategies combined descriptors related to nanocarbon materials, biointerfaces, biosensing, oncology, magnetic carbon systems, and translational validation, with terms adapted to the indexing conventions of each source.

Sources were screened for relevance to five domains: nanocarbon characterization and nano-biointerface behavior; biosensing mechanisms and analytical performance in biological matrices; magnetic carbon and hybrid systems; toxicological, manufacturing, and regulatory considerations; and comparator technologies for evaluating emerging magnetic nanocarbon platforms. Records were excluded when they were unrelated to biomedical translation, lacked adequate material or functional characterization, or advanced biomedical claims without experimental, clinical, regulatory, or comparator support. Non-peer-reviewed sources were included only when they consisted of official regulatory documents, recognized standards, or clinical registry records. Each retained source was classified according to the four evidence categories defined in Section 1.2. Adjacent nanocarbon and comparator magnetic studies were used to establish mechanistic plausibility, design requirements, and validation priorities, but not as direct evidence for a specific platform.

Clinical registry records were used only as contextual indicators of translational activity spanning the biosensing, biointerface, and magnetic-carbon domains addressed in this review. Among the retrieved ClinicalTrials.gov entries, four were retained as adjacent evidence. NCT07034248 describes the clinical translation of a label-free electrochemical biosensor based on nitrogen- and sulfur-doped graphene quantum dots decorated with gold nanoparticles for detecting breast cancer cells [22]. NCT07295340 evaluates folate-targeted near-infrared-II carbon dots as a fluorescent probe for ex vivo histopathological assessment of hepatocellular carcinoma [23].

NCT06368310, a first-in-human investigation, tested a graphene-based micro-electrocorticography array for cortical mapping during glioma surgery [24]. NCT06048367, a completed phase I trial, assessed intratumoral carbon-nanoparticle-loaded iron (CNSI-Fe(II)) in patients with advanced solid tumors [25]. These records identify neighboring directions of clinical activity but provide no direct evidence of biosensing accuracy, magnetic functionality, targeted delivery, or therapeutic efficacy for defect-associated or potentially metal-free magnetic carbon platforms.

## 3. Physicochemical Foundations of Nanocarbon Biomedical Interfaces

For biomedical applications, nanocarbons cannot be defined solely by their carbon composition. Composition provides only a chemical baseline, whereas functional performance is determined at the nano-biointerface. Structural and physicochemical attributes—including defect density, oxidation state, electronic structure, curvature, aggregation state, hydrodynamic size, colloidal stability, residual impurities, and surface functional groups—govern dispersion, biomolecular adsorption, recognition-element coupling, signal transduction, cellular uptake, tissue interactions, and immune responses. Consequently, carbon nanotubes, graphene oxide sheets, carbon dots, nanodiamonds, fullerenes, and defect-rich graphite fragments may share a carbon backbone while differing substantially in interfacial geometry, electronic structure, and biological identity [1,2,7,8].

**Figure 1:**
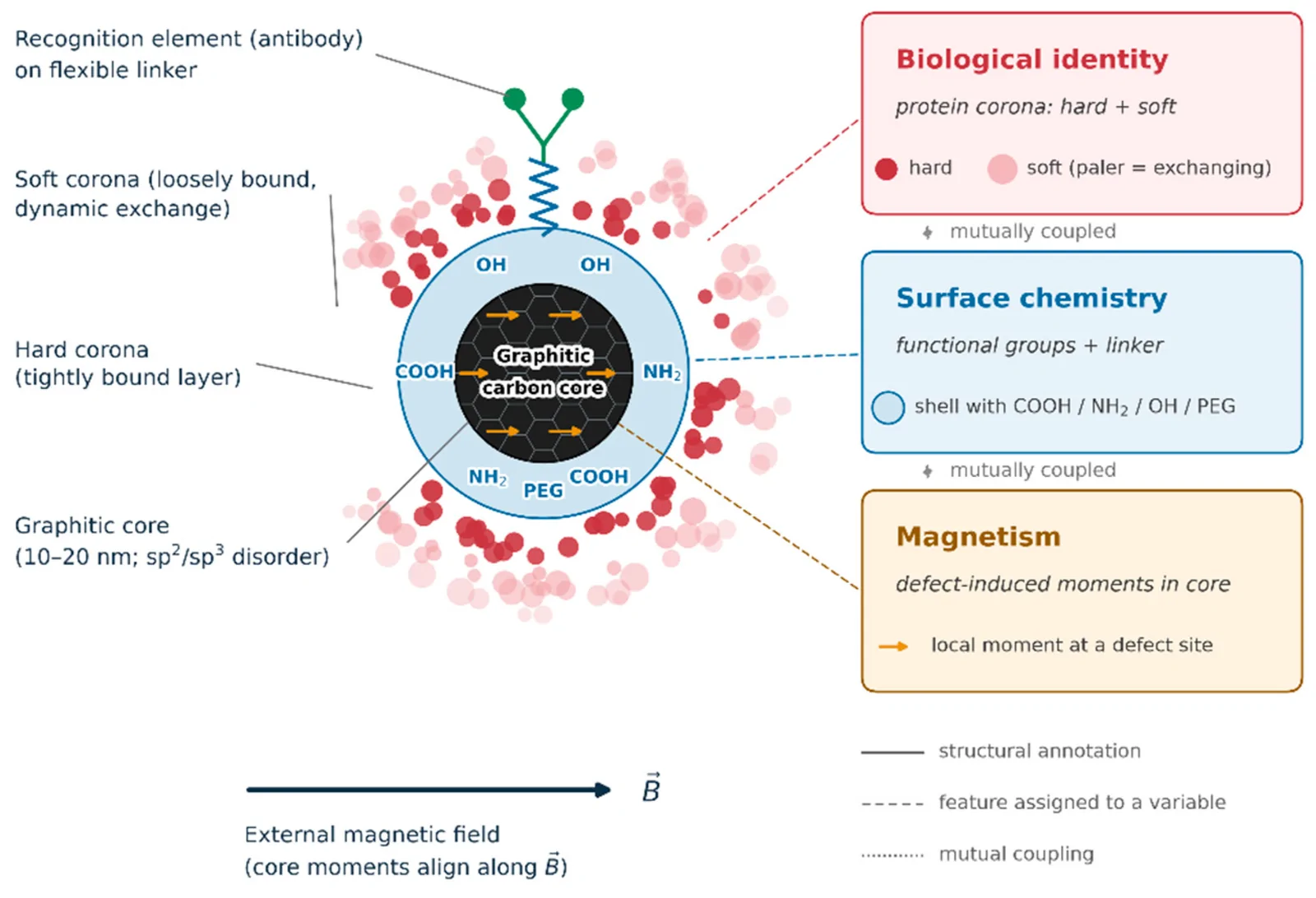
Schematic representation of an engineered magnetic carbon biointerface, highlighting the coupled relationships among the magnetic carbon core, surface chemistry layer, biomolecular corona, and recognition-element coupling. The scheme is conceptual and does not represent a validated clinical, diagnostic, therapeutic, or biosensing MAGUS platform.

Figure 1 illustrates this interface-centered perspective through a conceptual magnetic carbon biointerface. The scheme does not represent a single validated material; rather, it organizes the principal variables that define the biomedical identity of a magnetic carbon system, including a graphitic or defect-rich core, a surface-chemistry layer, an adsorbed biomolecular corona, a recognition element, and, where applicable, an external magnetic field. The figure emphasizes that magnetic behavior, surface chemistry, and biological identity are interdependent variables that require integrated characterization.

**Figure 2:**
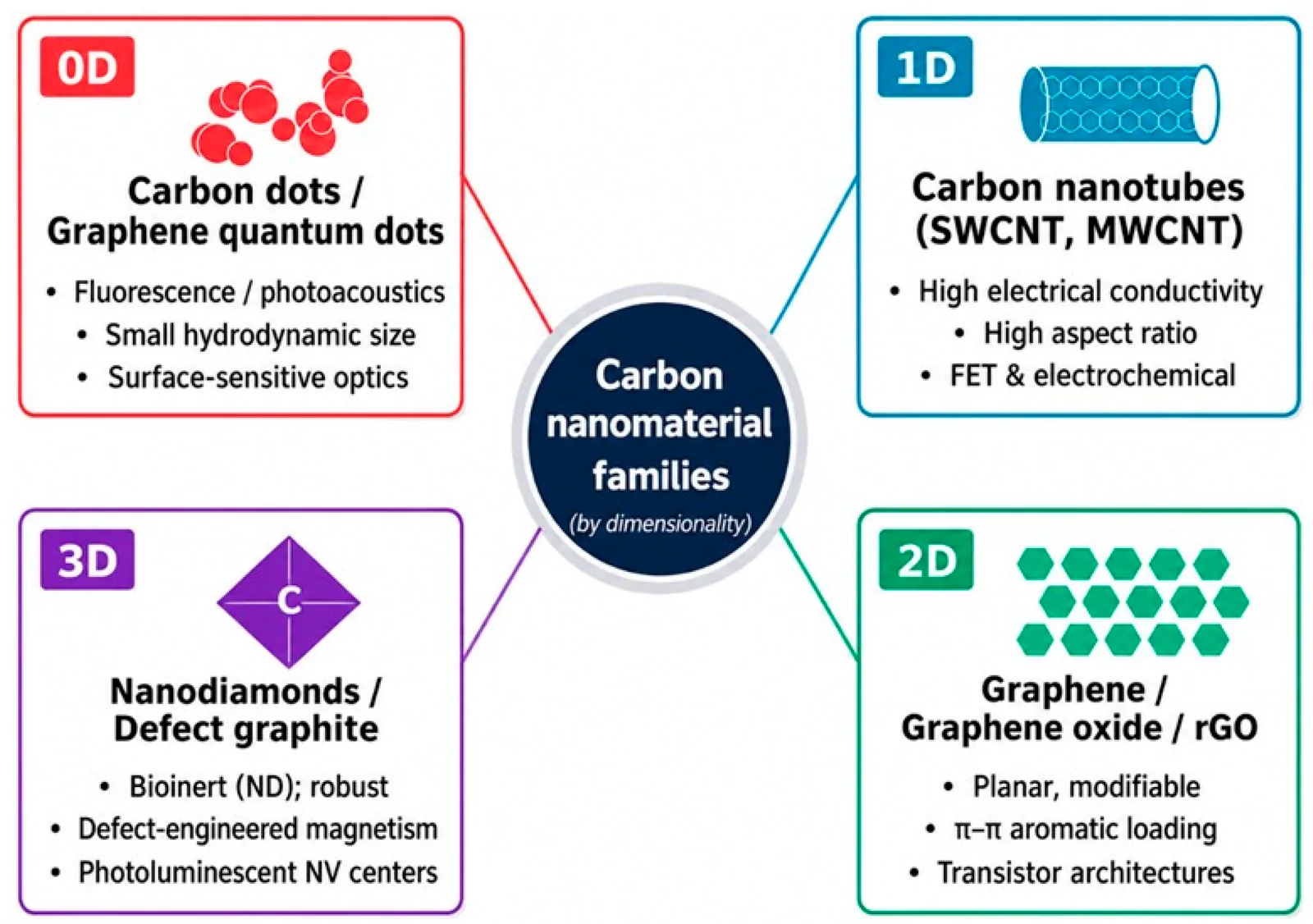
Conceptual classification of representative nanocarbon families by dimensionality and selected biomedical attributes. Class membership alone does not predict biomedical performance, which depends on surface chemistry, defect structure, purity, colloidal stability, functionalization strategy, biological matrix, dose, and intended application.

Dimensionality provides a useful initial classification, but it does not independently predict biomedical performance (Figure 2). One-dimensional carbon nanotubes provide conductive, high-aspect-ratio scaffolds for electrochemical and field-effect sensing. Two-dimensional graphene and graphene oxide offer planar, chemically modifiable surfaces for biomolecule immobilization, charge transfer, and transistor-based architectures. Zero-dimensional carbon dots and graphene quantum dots support optical, fluorescent, and electrochemical readouts that depend on particle size, surface states, heteroatom doping, and functional groups. Nanodiamonds and fullerenes are used in more specialized applications, including fluorescence imaging and radical scavenging. Across these material classes, biomedical relevance depends less on dimensional classification alone than on dispersion behavior, surface chemistry, dose, purity, matrix stability, functionalization reproducibility, and biological context [1,2,7,8].

The physicochemical behavior of magnetic nanocarbon systems is strongly influenced by structural defects, edge states, hybridization, heteroatom incorporation, adsorbed species, and residual impurities. Vacancies, chemisorbed hydrogen, sp^2^/sp^3^ disorder, heteroatoms, and edge-state density can modify the electronic and magnetic properties of carbon matrices, providing a theoretical basis for defect-engineered or defect-associated magnetism [26]. These same features may also affect charge transfer, electrochemical response, fluorescence, surface adsorption, and protein binding. However, physicochemical plausibility alone does not establish biomedical relevance. Translational viability requires reproducible synthesis, rigorous control and quantification of impurities, colloidal stability under physiologically relevant conditions, stable and reproducible surface chemistry, retention of function after bioconjugation and sterilization, and a safety profile appropriate to the intended application.

The biological environment can substantially modify the functional identity of nanocarbon. Upon contact with serum, plasma, interstitial fluid, mucus, or culture media, the material surface adsorbs proteins, lipids, salts, and metabolites, forming a biomolecular corona that may alter aggregation, recognition-element accessibility, receptor interactions, immune responses, biodistribution, cellular internalization, and fouling behavior. Protein-corona formation and biofouling should therefore be regarded as defining features of the nano-biointerface rather than as late-stage complications and should be incorporated into experimental design from the outset [27,28,29,30]. Molecular dynamics studies of peptide adsorption on graphene support this interpretation by showing that hydrophobic and π–π interactions can reorganize biomolecular conformations on carbon surfaces. Surface chemistry should therefore be considered not only a physicochemical property, but also a determinant of biological behavior [31].

These considerations apply directly to magnetic nanocarbon platforms, including potentially metal-free or defect-associated magnetic carbon architectures. In such systems, magnetic responsiveness, surface chemistry, and biological identity are functionally interdependent. Accordingly, claims involving magnetic guidance, analyte capture, imaging compatibility, or biosensing performance require direct validation under conditions that approximate the intended application. MAGUS-like candidates are considered part of this broader class and should be evaluated using the same criteria.

## 4. Nanocarbon Materials in Biosensing

Biosensing is among the most mature biomedical uses of nanocarbon materials. Carbon nanotubes and graphene-derived materials have been built into platforms of many kinds—electrochemical sensors, immunosensors and aptasensors, field-effect transistors, optical and fluorescence-based systems, and wearable or point-of-care formats [10,11,12,13,14,15,16,17,32]. What makes them useful is rarely a single intrinsic property; it is the convergence of several. Conductive carbon networks enhance charge transfer and signal transduction; large specific surface areas support high loading and the orderly placement of biorecognition elements; and tunable surface chemistry allows antibodies, aptamers, enzymes, nucleic acids, peptides, or molecularly imprinted layers to be immobilized to suit the analyte.

### 4.1. Electrochemical Nanocarbon Biosensors

Performance in buffer does not reliably predict performance in patient-derived matrices, where nonspecific adsorption, ionic-strength effects, redox-active interferents, protein adsorption, and recognition-layer instability may alter the analytical response. Electrochemical nanocarbon biosensors should therefore be assessed according to the broader validation criteria summarized in Table 1 and discussed in Section 4.5, including matrix testing, reproducibility, comparison with established methods, and alignment with a defined clinical use case [10,15,20,21].

The nanocarbon does more than enlarge the surface. Conductive graphitic domains shuttle electrons between the recognition layer and the electrode; oxygenated groups on graphene oxide or oxidized nanotubes covalently bind antibodies, aptamers, enzymes, peptides, or nucleic-acid probes; π–π interactions adsorb aromatic analytes and nucleic-acid sequences; and defect or edge sites govern electrocatalytic activity, background current, and interfacial charge distribution. Cancer targets span proteins, circulating nucleic acids, extracellular vesicle markers, metabolites, inflammatory mediators, and tumor-associated cell-surface antigens, so the interface must be matched to the analyte rather than assumed to be universal.

Sensitivity in a buffer does not predict performance in a patient sample. Serum, plasma, urine, saliva, and tissue lysates degrade a sensor through nonspecific adsorption, fouling, ionic strength effects, protein corona formation, redox-active interferents, and instability of the recognition layer. A device earns translational relevance only when its analytical reporting extends past the limit of detection to the full set of parameters and includes a direct comparison with an established method such as ELISA, PCR, immunohistochemistry, or imaging.

This priority, placing the use case before the material, mirrors the translation literature, which locates the failure of laboratory sensors not in their sensitivity but in manufacturability, workflow integration, reagent stability, standardized metrics, and regulatory readiness [20]. A liquid-biopsy screen, an intraoperative margin tool, a therapy monitor, and a recurrence-surveillance assay impose different sample volumes, thresholds, turnaround times, and validation cohorts; a platform designed without one of them in view rarely fits any.

Proof of concept marks the beginning of that path, not its end. Detecting a cancer biomarker on a nanocarbon electrode under controlled conditions demonstrates feasibility; it does not establish diagnostic accuracy, clinical utility, or superiority over existing tests. Translational maturity requires independent replication, real matrices, defined clinical endpoints, standardized fabrication and quality control, and comparison with reference methods [21].

For magnetic nanocarbon and MAGUS-like platforms, the electrochemical case is conditional on evidence that does not yet exist: stable coupling of the recognition element, retention of magnetic responsiveness in the working matrix, and reproducible integration onto the electrode, demonstrated together. The comparator systems reviewed here, including graphene, carbon-dot, magnetic-bead, and SPION electrodes, set the benchmark such a platform would have to meet; they do not stand in for it. The full validation requirements are set out in Section 4.5.

### 4.2. Optical and Fluorescent Carbon-Based Biosensors

Optical and fluorescent biosensors convert molecular recognition into changes in fluorescence intensity, wavelength, lifetime, or color, offering sensitivity, rapid response, and compatibility with miniaturized point-of-care formats. Carbon-based fluorophores have emerged as alternatives to conventional organic dyes and semiconductor quantum dots because they may offer improved photostability, aqueous compatibility, and reduced concerns about heavy-metal content in selected applications [31,32].

Carbon dots comprise a heterogeneous family of zero-dimensional carbon nanomaterials, generally below 10 nm in size, including carbon quantum dots, carbon nanodots, polymer dots, and graphene quantum dots. Their emission depends on particle size, crystallinity, surface defects, heteroatom doping, and surface functional groups. These features support tunable photoluminescence, aqueous dispersibility, comparatively favorable biocompatibility in selected experimental systems, photostability, and multiple conjugation sites for antibodies, aptamers, enzymes, and nucleic acids [31,32].

Graphene oxide and graphene quantum dots can perform complementary functions in fluorescence-based sensing. Graphene oxide commonly acts as an efficient quencher via electron transfer or Förster resonance energy transfer (FRET), whereas graphene quantum dots serve as fluorescent donors with size- and surface-dependent emission and relatively high photostability [33]. Their combination supports “on–off” and “on–off–on” architectures in which donor–quencher proximity suppresses emission and target recognition restores it [34].

FRET is one of the most widely used signal-transduction mechanisms in carbon-based fluorescence biosensors. Non-radiative energy transfer from an excited donor to an acceptor over approximately 1–10 nm depends on donor–acceptor distance, spectral overlap, dipole orientation, donor quantum yield, and fluorescence lifetime [35]. Together with photoinduced electron transfer and inner-filter effects, these mechanisms have been used to detect proteins, nucleic acids, enzymes, and extracellular vesicles [34,36].

Surface engineering remains a major determinant of analytical performance. Nitrogen doping may increase quantum yield, modify emission, lengthen fluorescence lifetime, and reduce non-radiative recombination, whereas amine, carboxyl, hydroxyl, and polymeric groups improve dispersibility and provide covalent attachment sites [31,32].

Kalkal et al. [37] illustrate this design in an oncologically relevant setting. Amine- and nitrogen-doped graphene quantum dots served as donors and gold nanoparticles as acceptors for neuron-specific enolase (NSE), a biomarker associated with small-cell lung cancer. Anti-NSE-functionalized dots emitted blue fluorescence; gold nanoparticles quenched the signal via nanosurface energy transfer; and NSE binding increased the donor–acceptor distance, thereby restoring emission. The assay responded within 16 min over the range 0.1 pg mL^−1^–1000 ng mL^−1^, achieved a reported limit of detection of 0.09 pg mL^−1^, and showed 94.69% recovery in serum [37].

Related fluorescence strategies have also been applied to extracellular vesicles and exosomes in liquid-biopsy workflows. FRET-based assays use graphene oxide, carbon nanotubes, and carbon dots as donors, acceptors, or quenchers to detect exosomal CD63, EpCAM, MUC1, PD-L1, and PSMA, as well as miR-21, miR-92a-3p, miR-133b, and miR-135b [36].

The principal translation limitation is not analytical sensitivity alone, but reproducibility and robustness in biologically complex fluids. Matrix quenching, autofluorescence, and inconsistent surface functionalization continue to separate reported proof-of-concept performance from routine assay operation, and relatively few studies benchmark their platforms against the clinical method they are intended to complement or replace [31,32,33].

### 4.3. Field-Effect and Transistor-Based Nanocarbon Biosensors

Field-effect transistors convert molecular recognition into changes in channel conductance, Dirac-point position, threshold voltage, or the full transfer curve. Graphene and carbon nanotubes are well suited to this architecture because of their high carrier mobility, conductivity, surface-to-volume ratio, and adaptable surface chemistry [38,39].

Graphene field-effect transistors (GFETs) provide a useful demonstration of label-free multiplexing. Guo et al. [40] detected hsa-miR-125b and Aβ42 from a single droplet on a 3 × 3 mm^2^ chip by using a hexamethyldisilazane blocking layer to spatially separate the two probe chemistries, reporting a detection range of 1 fM–100 pM without loss of specificity. Although the study addressed Alzheimer’s disease rather than cancer, it provides adjacent architectural evidence for label-free multiplexing. The design may inform oncology biosensor development, where combined biomarker panels are required to improve analytical discrimination across tumor types, disease stages, or treatment contexts; it should not, however, be interpreted as direct evidence in oncology.

The principal limitations of this format stem from the physical sensing environment and reproducibility. Debye screening at physiological ionic strength shortens the effective sensing distance and may reduce the response of devices that use large recognition elements such as antibodies. Nonspecific adsorption, biofouling, baseline drift, unstable functionalization, and device-to-device variability further complicate operation in serum, plasma, urine, saliva, or tissue extracts [39]. A credible GFET or nanotube-FET assay should therefore report functionalization reproducibility, transfer-curve stability, signal drift, selectivity against related biomarkers, performance at physiological ionic strength, recovery in spiked matrices, inter-device consistency, and comparison with an established analytical method [39].

For potentially metal-free or defect-associated magnetic carbon platforms, transistor-based sensing remains prospective. GFET and nanotube-FET studies provide device-design benchmarks, but platform-specific validation would require stable electrical behavior after functionalization and reproducible target recognition under realistic matrix conditions.

### 4.4. Magnetic-Assisted Biosensing and Preconcentration

Magnetic-assisted biosensing can improve workflow efficiency, analytical sensitivity, and selectivity by using magnetic materials as capture substrates, mobile reaction phases, separation tools, or labels. In complex oncology samples, low-abundance biomarkers may be masked by proteins, salts, lipids, cells, vesicles, and nonspecific adsorbates. A functionalized magnetic particle can bind the target, after which an external magnetic field enables separation, washing, preconcentration, and delivery of the captured complex to the detection zone [41,42].

The main analytical contribution of magnetic assistance lies in sample processing rather than in magnetism alone. Magnetic nanoparticles and beads functionalized with antibodies, aptamers, peptides, nucleic acid probes, enzymes, or other affinity ligands can isolate proteins, nucleic acids, extracellular vesicles, rare tumor cells, and related analytes prior to optical, electrochemical, fluorescent, impedimetric, or magnetic readout. Microchannel devices, lateral-flow formats, paper-based devices, and microfluidic systems use magnetophoretic trapping or field-assisted concentration to integrate incubation, washing, separation, and spatial control [41,42].

Magnetic enrichment can increase analytical availability, but it does not by itself establish diagnostic validity. Magnetic-assisted workflows should therefore be evaluated through capture efficiency, recovery after separation, nonspecific adsorption, washing efficiency, separation time, particle stability, ligand density, matrix tolerance, batch reproducibility, and compatibility with downstream readout [39,40,41]. For potentially metal-free or defect-associated magnetic carbon platforms, usefulness in capture and preconcentration would require these same metrics together with preserved magnetic responsiveness and stable carbon surface chemistry under realistic oncology conditions.

### 4.5. Analytical Validation Gaps: From Buffer Performance to Real Biological Matrices

A recurring weakness in nanocarbon biosensing is the gap between analytical performance under controlled conditions and performance in biologically complex matrices. Low limits of detection, broad linear ranges, and strong signal amplification are frequently reported in buffer or simplified spiked solutions, but these metrics may lose translational relevance when the assay is evaluated in serum, plasma, urine, saliva, cerebrospinal fluid, tissue lysates, extracellular vesicle preparations, or patient-derived samples. In these matrices, proteins, salts, lipids, metabolites, nucleases, proteases, cells, vesicles, redox-active molecules, and nonspecific adsorbates can shift the signal, block recognition sites, increase background, alter the nanocarbon biointerface, and reduce reproducibility [34,39].

A meaningful validation package must therefore extend beyond the limit of detection. For oncology biosensors, the minimum reporting set should include the target analyte, recognition element, nanocarbon component, transduction mode, biological matrix, limit of detection, limit of quantification, linear range, selectivity, recovery, reproducibility, stability, response time, interference testing, comparator method, and validation level. A sensor may be highly sensitive at trace concentrations yet saturate, drift, or lose proportionality across the clinically relevant range. The NSE assay discussed above illustrates a stronger reporting model because it includes response time, linear range, selectivity, reproducibility, and recovery in serum rather than relying on sensitivity alone [37].

Although the specific limitations vary according to the transduction mechanism, all platforms require validation under biologically relevant conditions. Electrochemical systems are particularly susceptible to fouling and electroactive interferents; optical platforms to autofluorescence and matrix quenching; field-effect devices to Debye screening and device-to-device variability; and magnetic-assisted systems to losses in capture efficiency, nonspecific binding, and particle instability [34,39,41,42]. These modality-specific limitations should be evaluated together with the common validation criteria summarized in Table 1.

Accordingly, comparator evidence derived from graphene, carbon nanotubes, carbon dots, magnetic beads, SPIONs, or magnetic graphene systems should be interpreted as design benchmarks rather than platform-specific validation. Early-stage defect-associated or potentially metal-free magnetic carbon platforms become analytically relevant only when direct experiments demonstrate stable functionalization, reproducible signal generation, matrix compatibility, preserved magnetic responsiveness, selective capture or recognition, and measurable analytical advantages over established workflows under defined oncology conditions. MAGUS-like candidates should be evaluated according to this same evidentiary standard.

## 5. Adjacent Nanomedicine Evidence and Implications for Biosensing Translation

Although this review focuses primarily on oncology biosensing, evidence from nanomedicine provides important contextual information for understanding the translational potential of nanocarbon platforms. Carbon nanomaterials are of particular interest because a single engineered architecture may, in principle, integrate biosensing, molecular imaging, drug delivery, and therapeutic functions. Within the scope of this review, however, nanomedicine studies are interpreted as adjacent evidence that informs the translation of biosensing rather than as direct validation of magnetic nanocarbon theranostic platforms [42].

Carbon dots and related nanocarbon systems illustrate this relationship particularly well. These materials have been investigated for fluorescence bioimaging, biosensing, multimodal MRI-, CT-, and photoacoustic-compatible imaging, photodynamic therapy, photothermal therapy, and integrated theranostic platforms [43,44,45,46,47]. Likewise, functionalization with targeting ligands, nuclear localization peptides, folate, hyaluronic acid, antibodies, and related biomolecules demonstrates how nanocarbon surfaces can be engineered to interact with specific biological targets. Nevertheless, the majority of these applications remain preclinical and therefore provide mechanistic and architectural guidance rather than direct evidence of clinical translation [43,44,45,46,47].

Demonstration of biological activity does not establish clinical applicability. Experimental studies may report improved imaging contrast, enhanced delivery, or photothermal and photodynamic antitumor effects, yet the biological behavior of the same material remains dependent on composition, dimensions, oxidation state, surface chemistry, purity, dose, route of administration, and biological context. Translation is further constrained by oxidative stress, inflammatory responses, genotoxicity, tissue accumulation, slow biodegradation, biomolecular-corona formation, off-target sequestration, incomplete clearance, and limited methodological harmonization across laboratories [41,42].

These limitations are directly relevant to biosensor development because the same surface and interfacial properties that control biodistribution, immune recognition, and cellular uptake also influence analyte accessibility, nonspecific adsorption, signal stability, and reproducibility in complex matrices. Nanomedicine evidence should therefore be used to define biointerface-related validation requirements rather than to imply that a material shown to image or treat a tumor will necessarily function as a reliable biosensor.

For defect-associated or potentially metal-free magnetic carbon systems, nanomedicine claims should remain provisional until platform-specific studies are available. Translational evaluation would require direct evidence of reproducible drug loading and controlled release, preservation of magnetic responsiveness after functionalization, colloidal stability and biomolecular-corona behavior under physiological conditions, hemocompatibility, predictable macrophage interactions, biodistribution, clearance, and measurable advantages over established nanocarriers. These requirements also provide relevant benchmarks for the translation of biosensing by testing whether the magnetic carbon biointerface remains stable, functional, and biologically interpretable under realistic conditions.

## 6. Magnetic Nanocarbon Platforms: Translational Landscape and Emerging Architectures

Magnetic nanocarbon platforms can be broadly classified into two categories that differ in both the origin of their magnetic behavior and their current level of translational maturity. The first comprises hybrid carbon-metal or carbon-metal oxide systems, including magnetic graphene oxide, carbon-coated magnetic nanoparticles, magnetic carbon nanotubes, magnetic carbon dots, graphitic nanocapsules, and carbon structures coupled to iron oxide. In these platforms, magnetic responsiveness is primarily provided by the established magnetic nanoparticle component, whereas the carbon phase contributes surface functionalization, payload loading, optical or electrochemical transduction, and processability. Collectively, these hybrid systems currently provide the strongest preclinical foundation for magnetic targeting, hyperthermia, molecular imaging, drug delivery, biosensing, analyte preconcentration, and multimodal theranostic applications [27,28,29,48,49,50].

The second category comprises defect-associated magnetic carbons, including ferromagnetic graphite, defect-engineered or irradiated graphene, heteroatom-doped carbons, and edge-state-rich, potentially metal-free carbon frameworks [19,26,51]. In these materials, magnetic behavior is attributed to vacancies, edge states, chemisorbed species, heteroatom incorporation, or sp^2^/sp^3^ disorder rather than to an intentionally introduced magnetic metal-oxide phase. Although this mechanism is physically plausible and supported by materials-science studies, it remains primarily a physicochemical proposition rather than an established biomedical function. Moreover, the absence of intentionally added magnetic metals does not imply biological inertness or eliminate concerns about aggregation, biomolecular corona formation, persistence, immune recognition, residual contamination, or application-dependent toxicity [26,51,52,53,54].

The distinction between these categories, therefore, reflects their stage of development rather than their terminology alone. Hybrid magnetic nanocarbons benefit from decades of advances in magnetic nanoparticle engineering, whereas defect-associated magnetic carbons must independently demonstrate comparable physicochemical robustness and application-specific performance. Required evidence includes rigorous magnetometry, quantification of residual metallic contamination, reproducible synthesis, colloidal and magnetic stability under physiologically relevant conditions, and preservation of magnetic responsiveness after functionalization. Where intrinsic or metal-independent magnetism is claimed, the observed response should also be shown not to result from uncontrolled metallic residues or catalyst contamination [26,51].

Biomedical utility therefore depends on more than the presence of a measurable magnetic moment. The magnetic response must be sufficient and stable for the intended operation, whether capture, enrichment, localization, tracking, or actuation, while aggregation, surface chemistry, batch variability, biological persistence, biodistribution, and safety remain controlled. These requirements are especially important because structural modifications introduced to improve dispersibility, ligand coupling, or biological compatibility may also alter electronic and magnetic behavior.

### Implications for Translational Development

Magnetic responsiveness provides a conceptual bridge between biosensing and nanomedicine, as magnetic fields can be used in both settings to modulate interactions at the nano-biointerface. In biosensing, magnetic materials may facilitate analyte capture, magnetic separation, washing, preconcentration, and transfer of target-bound complexes to electrochemical, optical, fluorescent, impedimetric, or magnetic readout systems [41,42]. In nanomedicine, related responsiveness may support localization, tracking, magnetic guidance, hyperthermia, or externally triggered functions, depending on platform design [28,55,56].

Evidence supporting one application, however, should not be interpreted as validation of another. Efficient magnetic capture within an analytical workflow does not establish therapeutic efficacy, imaging performance, biodistribution, or clinical readiness. Likewise, evidence of localization or imaging does not demonstrate biosensing selectivity or matrix-compatible analytical performance. Each proposed use therefore requires its own validation pathway, comparator technologies, performance metrics, and safety endpoints.

Novelty alone does not establish translational relevance. The key question is whether magnetic carbon architecture provides a measurable advantage over established comparator technologies, including SPIONs, magnetic graphene oxide, magnetic carbon nanotubes, magnetic beads, and non-magnetic nanocarbon biointerfaces. For biosensing, such an advantage may involve selective capture, efficient magnetic enrichment, reduced nonspecific adsorption, reproducible matrix-compatible functionalization, faster processing, or improved integration with an established readout. For delivery or theranostic applications, it may involve controlled loading and release, preserved magnetic responsiveness after surface modification, improved biodistribution, reduced off-target effects, or a more favorable safety-performance balance [41,42,55,56,57].

These advantages must be demonstrated through direct, head-to-head experiments under application-relevant conditions rather than inferred from material composition or theoretical functionality. Until such evidence becomes available, defect-associated or potentially metal-free magnetic carbon systems should be regarded as prospective magnetic nanocarbon architectures whose proposed biomedical functions have yet to be demonstrated experimentally.

## 7. Drug Delivery and Theranostic Applications as Adjacent Experimental Evidence

Drug delivery and theranostics are considered in this review as adjacent experimental evidence rather than as primary claims. Nevertheless, nanocarbon applications in these areas are not confined to theoretical design. Carbon nanotubes, graphene-derived materials, carbon dots, graphene quantum dots, and hybrid magnetic carbon systems have been synthesized, functionalized, loaded with therapeutic agents, evaluated in biological media, and tested in cellular and animal models. These studies demonstrate that nanocarbon architectures can support payload association, controlled or stimulus-responsive release, molecular imaging, photothermal and photodynamic effects, and multimodal therapeutic–diagnostic functions [44,45,46,47,48,49,50,51,57,58,59,60,61,62].

Their relevance to biosensing arises from the shared dependence on a stable and reproducible nano-biointerface. Drug delivery, imaging, and biosensing all require controlled surface chemistry, accessible functional groups, resistance to aggregation, predictable interactions with proteins and cells, and stable performance in biologically complex environments. Nanocarbons are particularly attractive in this context because their large specific surface area, tunable surface chemistry, π-rich structure, optical and electrochemical responsiveness, and multiple functionalization routes may support drug loading, ligand attachment, imaging-compatible tracking, and integration of diagnostic and therapeutic functions within a single architecture [44,47,57,59,62].

Experimental studies provide direct evidence that these functions can be achieved in specific nanocarbon systems. Carbon dots and graphene quantum dots have been investigated as fluorescence probes, multimodal imaging agents, drug carriers, photosensitizer-associated systems, and components of integrated theranostic platforms [45,46,47]. Gadolinium-doped carbon quantum dots, for example, have been developed as multimodal imaging materials, demonstrating that carbon-based nanostructures can be engineered to combine fluorescence with magnetic-resonance-compatible contrast [46]. Likewise, nucleus-targeting carbon nanoparticles have been investigated for tumor imaging and therapy through functionalization strategies designed to influence intracellular localization and biological interaction [47]. These findings confirm that nanocarbon theranostics have progressed beyond conceptual proposals, even though most remain at the physicochemical or preclinical stage.

Magnetic nanocarbon systems add another functional layer. Depending on composition and design, magnetic responsiveness may support field-assisted localization, magnetic enrichment, magnetic-resonance-compatible tracking, externally triggered functions, or spatial control of the nanomaterial–biological interface [28,29,48,49,50,55,56]. Magnetic graphene oxide, magnetic carbon nanotubes, and superparamagnetic carbon-based core–shell systems have been investigated for drug delivery, hyperthermia, imaging, and combined therapeutic applications [48,49,50]. In one representative example, superparamagnetic carbon multi-core shell nanoparticles were evaluated for doxorubicin delivery, including pH dependence, stability, and release kinetics [50]. Such studies provide direct experimental evidence for specific hybrid platforms, although they do not validate defect-associated or potentially metal-free magnetic carbons.

Carbon nanotube delivery studies further illustrate the experimental maturity and mechanistic depth achievable. PEGylated oxidized multi-walled carbon nanotubes have been examined as carriers for 5-fluorouracil, with loading and release assessed in relation to pH and surface chemistry [60]. Modified carbon nanotubes have also been studied for doxorubicin release, demonstrating that release behavior depends on protonation state, surface modification, and physicochemical interactions between the nanotube and the therapeutic molecule [61]. These studies move beyond theoretical loading capacity by quantifying payload behavior and identifying mechanistic variables that control release. However, they also show that successful loading in simplified conditions does not establish translational suitability.

The available literature therefore supports feasibility, but not a generalizable success rate. Outcomes vary with the carbon’s architecture, magnetic component, particle dimensions, oxidation state, surface chemistry, payload, route of administration, dose, disease model, comparator, and selected endpoint. A platform may generate strong fluorescence or magnetic-resonance contrast while providing limited therapeutic benefit; another may show high loading capacity but lose colloidal stability in serum; and a third may reduce tumor growth in an animal model while leaving long-term clearance, immunotoxicity, reproducibility, and manufacturability unresolved [44,57,58,59]. Magnetic nanocarbon delivery and theranostic systems should therefore be evaluated through defined performance criteria rather than broad claims of multifunctionality.

For drug-delivery applications, the relevant validation package includes payload identity, loading efficiency, batch-to-batch reproducibility, drug–carrier interaction, release kinetics, stability in biologically relevant media, preservation of magnetic or optical function after loading, biomolecular-corona formation, cellular uptake, intracellular localization, cytotoxicity, immune interaction, biodistribution, organ retention, clearance, and therapeutic efficacy under the intended route, dose, and disease context [52,53,54,57,58,59,60,61]. Demonstrating adsorption or release in buffer is therefore a necessary but insufficient step.

For theranostic applications, the evidentiary burden is even greater because the diagnostic and therapeutic components must both remain functional after integration. Imaging contrast, optical output, magnetic response, payload release, target recognition, and biological safety must be characterized in a single formulation. Evidence supporting one component cannot be assumed to validate the entire multifunctional system. A platform that performs effectively as an imaging probe may not provide adequate drug delivery, while a carrier with favorable release kinetics may not preserve optical or magnetic performance after functionalization.

Relevant comparator technologies include SPION formulations, magnetic graphene oxide, graphene oxide carriers, carbon-dot theranostics, liposomal drugs, polymeric nanoparticles, inorganic radiosensitizers, and porous silicon nanoparticles [55,56,57,58,59,62]. These comparators show that nanocarbon platforms are part of an active experimental field rather than a purely theoretical one. However, they also establish the benchmark that emerging magnetic carbon architectures must meet. A new platform would represent a non-incremental advance only if it demonstrated measurable advantages in loading reproducibility, colloidal stability in biological matrices, off-target toxicity, matrix tolerance, field-assisted localization, imaging–therapy integration, manufacturability, scalable functionalization, or overall safety–performance balance.

Accordingly, drug delivery and theranostics should be presented as experimentally supported but predominantly preclinical application domains. The literature demonstrates that nanocarbon biointerfaces can be engineered to perform concrete diagnostic and therapeutic functions, while simultaneously revealing the limitations that prevent automatic clinical extrapolation. For defect-associated or potentially metal-free magnetic carbon platforms, these studies establish mechanistic plausibility, identify comparator technologies, and define validation requirements, but they do not replace direct platform-specific evidence.

## 8. Translational Evaluation of Emerging Magnetic Nanocarbon Platforms: MAGUS as an Illustrative Case Study

The preceding sections established the physicochemical, analytical, biological, and translational criteria used in this review to evaluate magnetic nanocarbon platforms. The purpose of the present section is to apply this framework to emerging carbon magnetic architecture, using MAGUS exclusively as an illustrative prospective case study rather than as a validated biomedical, biosensing, therapeutic, or theranostic platform [19]. Accordingly, proposed applications—including tumor-biomarker detection, molecular imaging, CAR-T cell engineering, blood–brain barrier (BBB) transport or bypass, glioblastoma intervention, and potential advantages over established magnetic nanomaterials—are treated as application-specific hypotheses requiring direct experimental validation and comparative benchmarking [55,56,63].

Within this framework, translational maturity cannot be inferred solely from material novelty, magnetic behavior, or functionalization potential. Prospective magnetic nanocarbon platforms should instead be evaluated relative to technologies that have already reached different levels of experimental, preclinical, or clinical maturity. Relevant comparators include superparamagnetic iron oxide nanoparticles (SPIONs), magnetic graphene oxide, magnetic carbon nanotubes, magnetic beads, hybrid carbon–magnetic systems, defect-associated or potentially metal-free magnetic carbons, and clinically used carbon nanoparticle tracers [27,28,29,48,49,50,51,55,56,63,64,65,66,67]. These systems do not provide direct validation of MAGUS-like platforms, but they define realistic performance benchmarks, validation pathways, and translational endpoints.

The evidence landscape also demonstrates that translational readiness is application-dependent rather than a general property of the material. A platform validated for magnetic separation cannot automatically be regarded as suitable for drug delivery, imaging, cell therapy, or systemic administration. Similarly, evidence of tumor localization or therapeutic activity does not establish analytical selectivity, calibration stability, or reliable biosensing performance. Each intended use therefore requires its own characterization strategy, comparator technology, biological model, performance metrics, and safety assessment.

For biosensing, the critical requirements include reproducible recognition-element coupling, resistance to nonspecific adsorption and biofouling, analytical selectivity, clinically relevant limits of detection and quantification, calibration stability, batch and device reproducibility, and performance in complex biological matrices [20,39,41,42]. Magnetic-assisted formats require demonstration of capture efficiency, recovery after separation, washing performance, particle stability, ligand density, and preservation of magnetic responsiveness during assay operation.

Drug-delivery and theranostic evidence further illustrate why such distinctions are necessary. Nanocarbon and hybrid magnetic systems have experimentally demonstrated payload loading, controlled or pH-responsive release, fluorescence imaging, magnetic-resonance-compatible contrast, photothermal and photodynamic effects, and multimodal functions [44,45,46,47,48,49,50,51,57,58,59,60,61,62]. These findings confirm that the field extends beyond theoretical design. However, they remain specific to the material, formulation, payload, route, disease model, and endpoint under evaluation. Demonstration of drug adsorption, fluorescence emission, magnetic resonance contrast, or tumor growth inhibition in an experimental model does not establish reproducible delivery, clinical imaging performance, or therapeutic effectiveness of an emerging magnetic carbon architecture.

Accordingly, delivery and theranostic validation require direct assessment of loading efficiency, batch reproducibility, release kinetics, colloidal and functionalization stability, preservation of magnetic behavior after surface modification, biomolecular-corona formation, cellular uptake, immune interactions, biodistribution, clearance, and therapeutic benefit under the intended route, dose, and disease context [52,53,54,57,58,59,60,61]. SPION formulations, magnetic graphene oxide, graphene oxide carriers, magnetic carbon nanotubes, carbon-dot theranostics, liposomal drugs, polymeric nanoparticles, inorganic radiosensitizers, and porous silicon nanoparticles therefore serve as application-specific comparators rather than as substitutes for direct validation [55,56,57,58,59,60,61,62].

Cell-based applications introduce a distinct evidentiary burden. Magnetic functionalization of CAR-T or other immune cells must preserve viability, phenotype, proliferation, migratory behavior, cytokine production, antigen-specific cytotoxicity, and overall biosafety [63]. Evidence obtained with SPION-functionalized cells provides a relevant technological precedent, but it does not demonstrate that a magnetic carbon material will interact with therapeutic cells in the same manner.

Glioblastoma provides a particularly instructive example of why application-specific interpretation is essential. Passive BBB transport and BBB bypass through localized administration are fundamentally different strategies. Some magnetic systems are designed not to cross the BBB after systemic administration, but to circumvent it through intracranial or locoregional delivery. The magnetic continuum robot reported by Wu et al. accesses the brain through an Ommaya reservoir and delivers peptide nanophotosensitizers to intracranial tumors under magnetic guidance [68]. Magnetoelectric nanoparticles have likewise been investigated for field-controlled delivery of antitumor peptides to glioblastoma cells [69]. Neither approach demonstrates that a magnetic carbon platform intrinsically crosses the BBB, selectively accumulates in glioblastoma, or possesses intrinsic antitumor activity. Instead, these studies identify comparator strategies, experimental endpoints, and translational requirements against which a future magnetic nanocarbon platform would need to be assessed.

Table 2 summarizes this evidence landscape by positioning representative magnetic and carbon-based platforms according to their current evidence base, principal advantages, critical translational limitations, and relative maturity. The comparison is intended to illustrate a continuum of evidence rather than a fixed ranking of material quality. A platform may show advanced maturity in one localized application while remaining unsuitable or unvalidated for another.

### Translational Criteria for a Non-Incremental Magnetic Nanocarbon Platform

For a prospective magnetic nanocarbon platform to represent more than an incremental variation on existing technologies, it should demonstrate at least one reproducible, application-relevant advantage over established comparator systems. Such an advantage cannot be inferred solely from carbon composition, magnetic behavior, surface area, or theoretical multifunctionality. It must be shown experimentally under conditions that reflect the intended biomedical use.

Clinical registry records provide contextual evidence of neighboring translational activity but do not directly validate emerging magnetic carbon architectures. The retained records identify relevant clinical directions and endpoint structures but provide no evidence of biosensing accuracy, magnetic functionality, targeted delivery, or therapeutic efficacy for defect-associated or potentially metal-free magnetic carbon platforms [22,23,24,25].

For biosensing, potential non-incremental advantages include improved matrix tolerance, stronger antifouling performance, more efficient analyte capture and magnetic enrichment, lower nonspecific adsorption, greater functionalization reproducibility, faster sample processing, or superior integration with established analytical workflows. These gains should be demonstrated through head-to-head comparison with magnetic beads, SPION-based assays, graphene or carbon nanotube sensors, and validated electrochemical, optical, or field-effect platforms [15,20,39,41,42].

For delivery and theranostics, non-incremental value would require measurable improvement in loading reproducibility, controlled release, colloidal stability in biological fluids, preservation of magnetic and optical functions after functionalization, field-assisted localization, imaging–therapy integration, off-target toxicity, biodistribution, clearance, manufacturability, or overall safety–performance balance [55,56,57,58,59,60,61,62]. For cell-based applications, advantages must additionally be demonstrated without compromising cell viability, phenotype, proliferation, cytokine profile, migratory behavior, antigen-specific function, or safety [63].

Where potentially metal-free or intrinsic magnetic behavior is claimed, an additional evidentiary requirement applies. Magnetic characterization should be accompanied by rigorous quantification of metal and catalyst residues and, where technically possible, by evidence that the observed response cannot be explained by uncontrolled metallic contamination [26,51]. Magnetic behavior should also remain reproducible across batches and persist after dispersion, sterilization, bioconjugation, storage, and exposure to biologically relevant media.

Clinical registry records and clinical evidence from adjacent nanocarbon systems provide useful context but do not directly validate emerging magnetic carbon architectures. Carbon nanoparticle tracers, carbon-nanotube-enabled devices, graphene-derived systems, and hybrid nanocarbon–iron formulations identify neighboring areas of translational activity, accepted endpoints, and relevant comparator technologies [22,23,24,25,64,65,66,67,70]. They do not establish analytical accuracy, safety, efficacy, intrinsic magnetic behavior, or superiority for defect-associated magnetic carbon platforms.

Progression from an exploratory material to a translationally relevant platform therefore requires sequential validation. The material must first be defined through standardized physicochemical and magnetic characterization, impurity control, batch reproducibility, colloidal stability, sterilization compatibility, and storage stability. This should be followed by application-specific analytical or biological testing in relevant matrices and models, independent replication, direct benchmarking against established technologies, scalable manufacturing, and, where appropriate, regulatory evaluation.

Within the evidence framework adopted in this review, MAGUS remains an illustrative prospective case study because direct platform-specific biomedical validation has not yet been established. This classification reflects the current state of evidence rather than a demonstrated technological failure. Its progression toward translational relevance will depend on direct, application-specific experimentation and comparative benchmarking rather than extrapolation from adjacent nanocarbon or magnetic platforms.

## 9. Translational, Regulatory, and Industrial Barriers

What holds biomedical nanocarbon research back is not a lack of multifunctional materials—there are plenty. The hard part is turning these carbon architectures into systems that are reproducible, safe, scalable, sterilizable, clinically interpretable, and compatible with regulatory frameworks. Combining biosensing, imaging, delivery, and magnetic responsiveness only sharpens the problem, since every added function brings its own interacting demands on characterization, manufacturing control, validation, and risk assessment. Reproducibility, in particular, has to span the whole materials dossier—identity, impurity profile, surface chemistry, defect or graphitization state, colloidal behavior, and magnetic response under physiologically relevant conditions.

Manufacturing adds GMP-compatible production, sterilization, endotoxin control, storage and transport stability, byproduct control, and routine quality assays; the carbon-nanomaterial literature already identifies equipment and raw material costs, low yields, synthesis-route variability, and quality-control burden as practical barriers [57]. Critical quality attributes should include particle-size distribution, morphology, aggregation state, surface chemistry, zeta potential, defect or graphitization profile, metal or catalyst residues, endotoxin burden, sterility, colloidal stability in biological fluids, and application-specific magnetic or analytical performance.

A safety package of translational weight addresses hemocompatibility, immune activation, oxidative stress, genotoxicity, corona formation, biodistribution, organ retention, clearance, and chronic toxicity [52,53,54,57,58]—a profile that matters especially for magnetic nanocarbon, where magnetism, surface chemistry, aggregation, impurities, and biological persistence can interact in ways short-term in vitro assays miss. Regulatory translation is case-specific and cannot be read off material identity. Expectations shift with intended use, route, exposure duration, mode of action, and risk class: diagnostic use demands analytical performance, matrix-interference testing, reproducibility, stability, and clinical validation against comparators; therapeutic use demands chemistry, manufacturing, and controls, pharmacokinetics, biodistribution, toxicology, immunogenicity, dose justification, and demonstrated benefit; and combination or theranostic products carry the heaviest burden, since diagnostic, therapeutic, and device functions must be justified together. Current guidance reinforces this: FDA guidance on drug and biological products containing nanomaterials notes that such products may exhibit attributes distinct from conventional ones and warrant particular examination [71]; broader FDA nanotechnology guidance asks whether size-dependent properties were intentionally engineered, including outside the conventional 1–100 nm range [72]; and device-oriented evaluation follows the risk-management logic of ISO 10993-1 [73], reinforced by regulatory-science work on nanomaterial-based devices [74].

Multifunctionality, then, is as much an evidentiary obligation as a technological ambition. The most realistic near-term route is localized clinical translation, where exposure is bounded, endpoints are defined by the procedure, and interpretation is more direct than in systemic multifunctional nanomedicine [64,65,66,67,70]. For magnetic nanocarbon platforms, the sensible order is sequential: first the material itself—identity, magnetic behavior, impurity and colloidal control, sterilization, storage stability, and biological safety; only then application-specific performance measured against SPIONs, magnetic graphene oxide, carbon nanotube devices, carbon dot theranostics, magnetic bead assays, or standard diagnostic and therapeutic platforms.

## 10. Limitations of This Perspective

This is a critical narrative review rather than a systematic review, scoping review, meta-analysis, or formal technology assessment. The breadth of the topic allows for nanocarbon biosensing, nanomedicine, magnetic carbon systems, regulatory science, and oncology-oriented translation to be considered within a single framework, but it also limits how deeply each subfield can be examined.

A second limitation is intrinsic to the subject. Within the evidentiary scope of this review, MAGUS-specific biomedical validation is not yet available, and the platform’s description rests on a small number of reports by the present group [18,19]. The analysis, therefore, relies on adjacent evidence from nanocarbon materials, hybrid carbon–magnetic systems, clinical carbon tracers, magnetic nanoparticles, and regulatory frameworks. This approach is appropriate for framing hypotheses and selecting comparators, but it is not sufficient to support claims about diagnostic accuracy, therapeutic efficacy, clinical safety, manufacturability, or regulatory readiness.

A third limitation is temporal. Clinical-trial registry records and regulatory guidance can change. ClinicalTrials.gov entries and FDA documents should be reverified immediately before submission. Any changes in recruitment status, endpoints, regulatory language, or applicable standards should be reflected in the final manuscript.

## 11. Future Perspectives

Future work would be better off stopping the chase for multifunctionality for its own sake and instead benchmarking each proposed function against what already exists. A biosensing platform should be measured against graphene field-effect transistors, carbon nanotube sensors, magnetic bead-assisted assays, and validated electrochemical carbon interfaces. Delivery, theranostic, surgical-localization, and cell-therapy uses should be held against SPION-based formulations, magnetic graphene oxide, graphene oxide carriers, carbon-dot theranostics, carbon-nanoparticle tracer workflows, and SPION-functionalized immune-cell platforms.

Closing the gap between a material’s performance and clinical relevance will also require more biologically informative models. Tumor-on-chip systems, organoids, patient-derived models, immune-competent models, and complex biological matrices can stress-test nanocarbon platforms under conditions that more closely reflect human physiology. For biosensing, the next decisive step is likely to involve integration with microfluidics, wearable systems, multiplexed biomarkers, sample-processing modules, and point-of-care electronics, rather than continued emphasis on lowering detection limits in buffer. In nanomedicine, added value will need to be demonstrated relative to existing therapeutic and diagnostic technologies under comparable biological, manufacturing, and regulatory constraints.

## 12. Conclusions

Nanostructured carbon materials are important for oncology biosensing because the same low-dimensional traits—electronic structure and defect chemistry, optical behavior and conductivity, a functionalized surface, and interfacial adsorption—can be engineered to enable signal transduction and molecular recognition. Their quantum-relevant character is treated here as something that may shape electrochemical, optical, fluorescent, field-effect, and magnetic-assisted biosensing, never as a substitute for analytical validation. The real question is translational: whether a defined carbon biointerface can deliver reproducible, selective, matrix-compatible measurements against clinically relevant oncology targets.

Magnetic nanocarbon systems add field responsiveness to this already complex interface. Hybrid carbon–metal and carbon–metal oxide systems rest on a stronger preclinical and engineering foundation, particularly when the magnetic function is carried by well-characterized iron oxide components, whereas potentially metal-free or defect-associated magnetic carbons remain conceptually compelling but developmentally earlier.

A critical framework has to hold three lines steady: between demonstrated capability and projected functionality, between adjacent evidence and platform-specific validation, and between an architectural hypothesis and an experimentally grounded performance claim. Held to those lines, MAGUS can serve as a strategic hypothesis for magnetic nanocarbon biointerfaces—provided the account remains comparative, evidence-bound, and anchored in the data that actually exist.

The immediate priorities are concrete: define the material, control its impurities, establish biointerface stability and safety in complex biological matrices, and test it head-to-head against SPIONs, magnetic graphene oxide, magnetic carbon nanotubes, carbon-dot theranostic systems, and nonmagnetic nanocarbon biointerfaces. What the field lacks is not another platform announced as transformative but the rigorous testing that would show which platforms actually translate.

## Figures and Tables

**Table 1:** Minimum analytical information expected for electrochemical nanocarbon biosensors in oncology-related applications.

Minimum Expectation for Translational Relevance	Why It Matters	Parameter
Clearly specified cancer biomarker, molecular class, or cellular target	Defines the clinical or biological purpose	Target analyte
Antibody, aptamer, peptide, enzyme, nucleic-acid probe, or molecularly imprinted layer	Determines analytical specificity	Recognition element
Graphene, GO, rGO, CNTs, carbon dots, GQDs, nanodiamonds, or a hybrid carbon system	Defines the interfacial and transduction role	Nanocarbon component
Voltammetric, amperometric, impedimetric, potentiometric, or conductometric readout	Defines signal generation	Transduction mode
Serum, plasma, urine, saliva, tissue lysate, extracellular-vesicle preparation, or patient-derived sample	Determines real-world relevance	Biological matrix
LOD, LOQ, linear range, selectivity, recovery, reproducibility, stability, and response time	Determines measurement quality	Analytical performance
Testing against nonspecific proteins, salts, metabolites, redox-active molecules, and related biomarkers	Determines matrix robustness	Interference control
ELISA, PCR, imaging, immunohistochemistry, a laboratory assay, or an established sensor platform	Determines clinical interpretability	Comparator method
Buffer-only testing, spiked matrix, real sample, patient cohort, independent replication, or clinical validation	Defines evidence maturity	Validation level

**Table 2:** Comparative evidence-boundary matrix for MAGUS and magnetic/carbon-based comparator platforms.

Platform	Current Evidence	Potential Advantage	Critical Translational Limitation	Maturity Level
SPIONs	Broad preclinical foundation and established biomedical use in selected imaging, targeting, hyperthermia, and cell-interface applications	Magnetic manipulation, imaging support, hyperthermia, and established functionalization routes	Aggregation, formulation-dependent safety, metabolism, biodistribution, and clearance	High
Magnetic graphene oxide	Moderate preclinical evidence in loading, magnetic manipulation, imaging, photothermal applications, and interfacial engineering	Combination of therapeutic loading, carbon surface chemistry, and magnetic or photothermal response	Surface heterogeneity, colloidal instability, reproducibility, toxicology, and manufacturing control	Moderate
Magnetic carbon nanotubes	Limited experimental and preclinical evidence	Conductivity, high surface area, payload loading, and magnetic responsiveness	Fibrous morphology, persistence, aspect-ratio-dependent toxicity, impurities, and reproducibility	Low–moderate
Defect-associated or potentially metal-free magnetic carbon	Fundamental and emerging materials-science evidence	Potential magnetic responsiveness without an intentionally introduced metal-oxide core	Need to establish intrinsic magnetic origin, exclude metallic contamination, and demonstrate biological performance	Low
Carbon nanoparticle surgical tracers	Prospective and randomized clinical evidence in selected lymph-node localization and staging workflows	Procedure-controlled localization and clinically interpretable endpoints	Localized tracer role; no evidence of systemic magnetic control, delivery, biosensing, or platform-wide efficacy	Moderate–high for localized use
MAGUS	Conceptual and prospective evidence based on a limited number of reports	Prospective magnetic carbon architecture with potential biointerfacial functionalization	No direct biosensing, therapeutic, imaging, safety, or manufacturing validation	Exploratory

Note. Maturity levels are qualitative and reflect the relative strength and application-specific scope of the available biomedical evidence. “High” indicates established biomedical use in selected applications; “moderate–high” indicates clinical use under localized or procedure-controlled conditions; “moderate” indicates a broader preclinical foundation with unresolved translational constraints; “low–moderate” and “low” indicate limited or emerging experimental evidence; and “exploratory” indicates that direct platform-specific biomedical validation remains unavailable within the evidentiary scope of this review.

## Data Availability

No new data was generated in this study. All sources informing this critical narrative review are cited and publicly available through the indicated DOIs, journal websites, or regulatory agency repositories.

## Abbreviations

The following abbreviations are used in this manuscript:

MAGUS	Magnetic Graphite Universal System
SPIONs	Superparamagnetic Iron Oxide Nanoparticles
FDA	U.S. Food and Drug Administration
ISO	International Organization for Standardization
MRI	Magnetic Resonance Imaging
PET	Positron Emission Tomography
ELISA	Enzyme-Linked Immunosorbent Assay
PCR	Polymerase Chain Reaction
GO	Graphene Oxide
rGO	Reduced Graphene Oxide
CNTs	Carbon Nanotubes
GQDs	Graphene Quantum Dots
LOD	Limit of Detection
LOQ	Limit of Quantification
FRET	Förster Resonance Energy Transfer
NSE	Neuron-Specific Enolase
CD63	Cluster of Differentiation 63
EpCAM	Epithelial Cell Adhesion Molecule
MUC1	Mucin 1
PD-L1	Programmed Death-Ligand 1
PSMA	Prostate-Specific Membrane Antigen
miR	microRNA
GFETs/GFET	Graphene Field-Effect Transistor(s)
FET	Field-Effect Transistor
DNA	Deoxyribonucleic Acid
RNA	Ribonucleic Acid
CT	Computed Tomography
CAR-T	Chimeric Antigen Receptor T Cell
BBB	Blood–Brain Barrier
HPLC	High-Performance Liquid Chromatography
GMP	Good Manufacturing Practice
CAPES	Coordenação de Aperfeiçoamento de Pessoal de Nível Superior
IATQ	Inteligência Artificial e Tecnologias Quânticas
FINEP	Financiadora de Estudos e Projetos
